# Cholesterol: Its Regulation and Role in Central Nervous System Disorders

**DOI:** 10.1155/2012/292598

**Published:** 2012-10-17

**Authors:** Matthias Orth, Stefano Bellosta

**Affiliations:** ^1^Institut für Laboratoriumsmedizin, Vinzenz von Paul Kliniken gGmbH, Adlerstra**β**e 7, Postfach 103163, 70199 Stuttgart, Germany; ^2^Dipartimento di Scienze Farmacologiche e Biomolecolari, Facoltà di Farmacia, Università di Milano, Via Balzaretti 9, 20133 Milano, Italy

## Abstract

Cholesterol is a major constituent of the human brain, and the brain is the most cholesterol-rich organ. Numerous lipoprotein receptors and apolipoproteins are expressed in the brain. Cholesterol is tightly regulated between the major brain cells and is essential for normal brain development. The metabolism of brain cholesterol differs markedly from that of other tissues. Brain cholesterol is primarily derived by *de novo* synthesis and the blood brain barrier prevents the uptake of lipoprotein cholesterol from the circulation. Defects in cholesterol metabolism lead to structural and functional central nervous system diseases such as Smith-Lemli-Opitz syndrome, Niemann-Pick type C disease, and Alzheimer's disease. These diseases affect different metabolic pathways (cholesterol biosynthesis, lipid transport and lipoprotein assembly, apolipoproteins, lipoprotein receptors, and signaling molecules). We review the metabolic pathways of cholesterol in the CNS and its cell-specific and microdomain-specific interaction with other pathways such as the amyloid precursor protein and discuss potential treatment strategies as well as the effects of the widespread use of LDL cholesterol-lowering drugs on brain functions.

## 1. Introduction

Cholesterol is an important structural component of cellular membranes and myelin and a precursor of oxysterols, steroid hormones, and bile acids. Cholesterol is a major constituent of the human brain (with about 35 grams of cholesterol in an adult brain [1]), and the brain is the most cholesterol-rich organ [2], containing about 20% of the body's total cholesterol. Brain lipids consist of glycerophospholipids, sphingolipids, and cholesterol in roughly equimolar proportions [3]. Cholesterol is tightly regulated between the major brain cells—neurons and glia, that is, astrocytes, microglia, and oligodendrocytes—and is essential for normal brain development. Cholesterol is required for synapse and dendrite formation [4, 5], and for axonal guidance [6]. Cholesterol depletion leads to synaptic and dendritic spine degeneration, failed neurotransmission, and decreased synaptic plasticity [7]. Cholesterol is a pivotal constituent of cell membranes, steroid hormones, and for the function of the hedgehog protein [8]. Defects in cholesterol metabolism lead to structural and functional central nervous system (CNS) diseases such as Smith-Lemli-Opitz syndrome [9], Niemann-Pick type C (NPC) disease [10], Huntington's disease [11], and Alzheimer's disease [12]. These metabolic defects affect different metabolic pathways such as (1) cholesterol biosynthesis, (2) lipid transport and lipoprotein assembly, (3) receptors that mediate the cellular uptake of lipids, and (4) signaling molecules [13].

Unlike cholesterol in other organs in the periphery, brain cholesterol is primarily derived by *de novo* synthesis. The intact blood brain barrier (BBB) prevents the uptake of lipoproteins from the circulation in vertebrates [14]. In cells outside the brain, the need for cholesterol is covered by uptake of lipoprotein cholesterol by cells as well as by *de novo* synthesis [15]. The importance of this isolated cholesterol pool in the CNS has been described as early as 1834 when Couerbe called cholesterol “un element principal” (a key element) of the CNS [16]. Cholesterol is synthesized via the isoprenoid biosynthetic pathway (for further details see [17]). Isoprenoid biosynthesis starts with acetyl-CoA as a substrate, which by means of 6 subsequent enzyme reactions is converted into isopentenyl-pyrophosphate, the basic C_5_ isoprene unit used for synthesis of all subsequent isoprenoids. In total, at least 20 enzymes are involved for the generation of cholesterol [17]. The 3-hydroxy-3-methylglutaryl-coenzyme A reductase (*Hmgcr*; EC 2.3.3.10) is the rate limiting enzyme in cholesterol biosynthesis and the target of statin pharmacotherapy [18]. Besides cholesterol, the cholesterologenic pathway forms other important intermediates such as mevalonate, farnesyl pyrophosphate, squalene, and lanosterol [19]. The first enzymes of the isoprenoid/cholesterol biosynthetic pathway, that is, the conversion of acetyl-CoA to farnesyl pyrophosphate, are localized in the cytosol except for *Hmgcr *which, together with most enzymes involved in cholesterol synthesis, is localized in the endoplasmatic reticulum [20, 21].

In contrast to the distribution in plasma lipoproteins, essentially all (>99.5%) cholesterol in the CNS is present in an unesterified, free form [2]. There are two major pools of CNS cholesterol: one pool (containing up to 70% of the CNS cholesterol) consists of the myelin sheaths of oligodendroglia; the other pool is made up by plasma membranes of astrocytes and neurons. The lipid-protein composition of myelin differs from that of other cell membranes; myelin dry weight consists of about 70% lipids and 30% proteins, in other cell membranes the distribution is about 30% lipids and about 70% proteins [2]. Major lipid constituents of myelin are cholesterol, phospholipids, and glycosphingolipids in molar ratios of about 4 : 4 : 2. The lipid composition, in particular the cholesterol composition of myelin, is believed to have a pivotal role in membrane morphology and function such as the transmission of nerve impulses. In neurons (which are composed of cell body and axon), electrical impulses are transmitted rapidly along the axon. The axon is wrapped by myelin made up from the membranes of several oligodendrocytes, separated by periodic gaps in the myelin sheath—called nodes of Ranvier. This discontinuous insulation allows the saltatory conduction of the action potential. Due to its reduced permeability to ions, the cholesterol enrichment of the myelin sheath propagates the transmission of current along the axon rather than across the membranes of oligodendrocytes [22]. 

## 2. Brain Cholesterol

Brain cholesterol is considered to be a distinct pool from body cholesterol. Unlike cholesterol in other organs, there is only minute exchange of cholesterol towards other organs (2). The majority of brain cholesterol accumulates between the perinatal period and adolescence when neurons are encircled by specialized plasma membranes termed myelin. After myelination, the metabolism of cholesterol in the adult brain is characterized by a very low turnover and minimal losses [23]. However, recent results indicate that both cholesterol synthesis and degradation are active in the adult brain as well and that alteration in these mechanisms profoundly influences higher-order brain functions [24]. 

Several levels of evidence indicate a distinct metabolism of CNS cholesterol. The brain does not respond to many control mechanisms operative in maintaining cholesterol homeostasis in the whole organism. The half-life of brain cholesterol in the adult organism is between 6 months and 5 years [25, 26], the half-life of plasma cholesterol, in contrast, is only a few days [27]. In the 1940s, administration of deuterated water into rats led to an incorporation of the label into the unsaponifiable lipids of the brain [28]. Similar findings were observed in unrelated studies employing deuterated cholesterol in dogs [29]. From the current level of knowledge, in the CNS of mammals such as mice, rats, and humans, >95% of cholesterol is synthesized *de novo* from acetate and exchange between plasma lipoprotein cholesterol and brain cholesterol has only very little impact [1, 30–33]. However, some studies could demonstrate the transfer of small amounts of cholesterol from the periphery through the BBB to the CNS. In humans, after administration of 4-^14^C-labelled cholesterol, on average 3.2% of the serum label was detected in the CNS cholesterol [34]. A drawback of this study is the fact that all patients were terminally ill (most of them with malignancies) and a defective BBB could not be excluded. No label was detected within the first days after injection and the authors described the cholesterol exchange as negligible. The accumulation of minute amounts of cholesterol was also observed in adult guinea pigs after administration of hexadeuterium-labelled cholesterol with an accumulation of 1.23% and 0.93% in the cerebrum and cerebellum, respectively [35], and in mice and rats after feeding a diet containing hexadeuterium-labelled cholesterol with an accumulation of less than 1% [36].

Recent data indicate that besides being an important constituent of the brain cell membranes, cholesterol as well as the receptors for cholesterol-containing molecules are pivotal signaling molecules for brain morphology during embryonic development. During embryonic development and in the early postnatal period, the central nervous system undergoes an enormous cellular expansion. A sufficient availability of cholesterol as well as a correct ratio of cholesterol and phospholipids is crucial for the physicochemical properties of cells within the CNS. It is conceivable that during evolution a checkpoint was established that determines which sufficient cholesterol is available to proceed with the expansion of the brain or whether cell division and growth are slowed down [37]. The highest rate of cholesterol synthesis in humans and rodents occurs during the first postnatal weeks [30, 38, 39]. This time window corresponds with the peak of the myelination process and the myelination process is delayed when cholesterol biosynthesis is deficient [40].

## 3. Sterol Flux between CNS Cells

A sufficient availability of cholesterol is necessary for normal neuronal function and morphology, and both a lack and surplus of cholesterol impair these features [41, 42]. Cholesterol in neurons can be synthesized by neurons themselves [43, 44] and can also be taken up from other cells within the CNS, namely, from oligodendrocytes [45]. Oligodendrocytes have a central role in cholesterol synthesis in the CNS. On the contrary, the role of neurons and glial cells in cholesterol biosynthesis is still poorly understood [46]. Data indicate that neurons (in particular regions of the brain and/or under certain conditions) synthesize and take up cholesterol from circumjacent oligodendrocytes. Enzymes such as *Hmgcr* and 7-dehydrocholesterol reductase (*Dhcr7; EC 1.3.1.21) *are expressed with high transcript levels in cortical, cholinergic, and hippocampal neurons [47]. The expression of cholesterol-synthesizing enzymes as well as sterol-sensing factors, intracellular transporters, cholesterol shuttle proteins, and lipoprotein receptors is different within regions of the brain [13, 48]. Consequently, different regions of the brain differ markedly in their cholesterol content [49].

During maturation of neurons, the endogenous synthesis of cholesterol is impaired and the neurons depend on cholesterol provided by astrocytes [50]. Brain-derived neurotrophic factor (BDNF) is an important stimulus for *de novo* synthesis of cholesterol in neurons [51]. The significant transfer and uptake of oligodendrocytes-derived cholesterol by neurons could be demonstrated by conditional ablation of cholesterol synthesis in mice neurons. The conditional gene inactivation of the squalene synthase gene (*fdft1*) in neurons revealed a normal phenotype and function [45]. This supports the hypothesis that significant amounts of cholesterol are transferred between different cell types in the CNS.

The transfer of cholesterol between different cells is influenced by the fluidity of cell membranes and the distribution of microdomains such as lipid rafts (also known as detergent-resistant membrane fraction (DRM)). One example for a disease with altered composition of cell membranes is the deficiency of *Dhcr7. *Total or partial deficiency of *Dhcr7* causes Smith-Lemli-Opitz syndrome with the clinical triad developmental deformities, incomplete myelination, and mental retardation [52]. Tissue cholesterol and total sterol levels are markedly reduced, and 7-dehydrocholesterol levels are highly elevated [53]. High concentration of 7-dehydrocholesterol inhibits *Hmgcr* which exacerbates the cellular cholesterol deficit [54]. The altered membrane composition, in particular the increased 7-dehydrocholesterol levels, increases membrane fluidity [55] and decreases the intermolecular packing of phospholipid fatty acyl chains [56, 57]. The altered membrane composition leads to functional changes. The depletion of cholesterol, followed by replacement by 7-dehydrocholesterol in hippocampal membranes, does not restore the ligand-binding of the serotonin 1A receptor [58]. 

Abnormalities in cholesterol homeostasis have been also observed in Huntington's disease [59] and stress the necessity of cholesterol transfer within cells of the CNS (i.e., between the major site of cholesterol biosynthesis—oligodendroglia cells—and neurons). Transcription of genes in the cholesterol and fatty acid biosynthetic pathways are downregulated in human postmortem Huntington's disease striatal and cortical tissues as well as in murine models of Huntington's disease [60]. The hallmark of Huntington's disease, the accumulation of mutant huntingtin protein within neurons, is affected by the inefficient palmitoylation of huntingtin. This palmitoylation is crucial for the normal function since this process enhances the hydrophobicity of this protein and determines the membrane association and the subcellular trafficking between membrane domains [11, 61]. The accumulation of cytoplasmic and nuclear inclusions leads to neuronal dysfunction and later to neuropathological changes such as cell loss and atrophy in the putamen and neostriatum.

## 4. Net Sterol Flux of CNS Cholesterol

**Figure 1:**
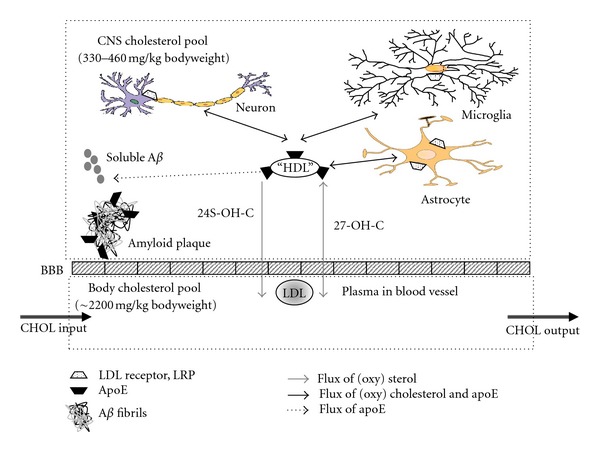
Major cholesterol and apoE pathways in the CNS. Cholesterol is synthesized de novo in brain cells (neurons, astrocytes, microglial cells). Efflux of CNS cholesterol through the BBB occurs as 24(S)-hydroxycholesterol (24S-OH-C) and 27-hydroxycholesterol (27-OH-C). 24S-OH-C is produced exclusively in the CNS, 27-OH-C is produced in most organs. Unlike cholesterol, 24-S-OH-C and 27-OH-C can cross the BBB because of the hydroxylated side chains. Primarily astrocytes and microglia secrete HDL-like lipoproteins composed of cholesterol and phospholipids and apoE as the major apoprotein. ApoE is the ligand of these lipoproteins to the receptors of the LDL receptor family such as the LDL-receptor and LRP. Exchange of cholesterol and apos between CNS cells occurs via these lipoproteins. In plasma, 24S-OH-C and 27-OH-C are transported on lipoproteins such as LDL and HDL. *De novo* cholesterol synthesis in CNS cells can be regulated by the apoE-mediated uptake of lipoproteins via the LDL receptor family. ApoE is produced within the CNS and interacts with A*β*. The availability of cholesterol and of apoE are thought to affect amyloidogenesis and apoE (in particular the isoform apoE4) promoting the formation of amyloid fibrils from soluble A*β* in the CNS. The data for the steady state cholesterol pool have been determined from studies in healthy adults. The flux of cholesterol across the whole body is ~700 mg/day (CHOL INPUT/OUTPUT). The flux across the CNS is only 0.9% of whole body (~12 mg/day). The efflux of 24(S)-hydroxycholesterol through the BBB is limited to ~6-7 mg per day [64, 67], the daily influx of 27-hydroxycholesterol into the brain has been estimated to be ~5 mg [1]. Please note that the brain per kg organ contains 10 times more cholesterol than the rest of the body.

Most of the CNS cholesterol is recycled. However, mechanisms to export cholesterol into the circulation are mandatory to maintain homeostasis. Two different pathways for exporting cholesterol have been identified so far. Similar to other cells such as macrophages, cells of the CNS, in particular astrocytes, shed cholesterol associated with apolipoprotein (apo) E into the cerebrospinal fluid (CSF). Despite lipoproteins in the brain are secreted predominantly by glia cells, neurons are also capable of synthesizing lipoproteins under certain conditions [62, 63]. However, the capacity of the shedding pathway is very limited and can export only 1-2 mg cholesterol per day [14]. The second, quantitatively more important mechanism is the export of cholesterol as 24(S)-hydroxycholesterol. Unlike nonoxydized cholesterol, oxysterols such as 24(S)-hydroxycholesterol can cross lipophilic membranes such as the BBB at a much faster rate than cholesterol itself [64, 65]. The introduction of a hydroxyl group into the side chain of cholesterol leads to a local reordering of membrane phospholipids that is energetically more favorable and allows a transfer of oxidized cholesterol through the membrane several orders of magnitude greater than that of nonoxidized cholesterol [66] (Figure 1). 

The flux of 24(S)-hydroxycholesterol through the BBB is limited to about 6-7 mg per day [64, 67].

In the circulation and in most tissues, cholesterol is always present in a great excess compared to oxysterols with the ratio of cholesterol to any oxysterol being 1.000 : 1 to 100.000 : 1 [68]. In the brain, however, the ratio is much lower and varies between 500 : 1 and 1000 : 1 [67]. Oxysterols can shuttle between the membrane leaflets and can be extracted by acceptors such as lipoproteins. Plasma oxysterols are associated with membranes and in plasma are bound to lipoproteins [69], similar to other lipids present in trace amounts such as gangliosides [70, 71]. Similar to the ganglioside/cholesterol ratio [70], the ratio of 24(S)-hydroxycholesterol to cholesterol in plasma is rather constant and patients with hypercholesterolemia show higher plasma concentration of 24(S)-hydroxycholesterol [68]. 

While in the brain the oxidation of the steroid side chain at position 24 is the primary mechanism for the elimination of cholesterol, outside the brain the oxidation occurs at position 27. The enzyme responsible for this reaction is sterol 27-hydroxylase (27-OHC, CYP27A1) and macrophages have particularly high activities of CYP27A1 [68]. 27-hydroxycholesterol is able to pass the BBB [65] and the daily influx of 27-hydroxycholesterol into the brain has been estimated to be 5 mg. This flux is dependent on the concentration in the circulation and on the integrity of the BBB [72]. The rate limiting step in bile acid synthesis is 7*α*-hydroxylation [73]. In the liver, 7*α*-hydroxylation of cholesterols is mediated by CYP7A and CYP39A1 while in brain and other tissues, both sterols and some steroids including dehydroepiandrosterone are prominently 7*α*-hydroxylated by CYP7B [73]. In human plasma, 7-hydroxycholesterol is the quantitatively most prevailing oxysterol [69]. 

In the total organism, the pool of cholesterol is about 2200 mg/kg body weight and similar in most species. However, the cholesterol pool in the CNS varies from about 330 mg/kg body weight in the mouse up to 460 mg/kg body weight in primates [27]. In humans, the flux of cholesterol across the whole body is about 10 mg/day per kg body weight while the flux across the CNS is 0.09 mg/day per kg body weight only [64] (Figure 1). In smaller animals such as the mouse and the baboon, the rates are several folds higher. It is remarkable that in all species studied so far, the percentage of the rate of cholesterol flux across the CNS is about 0.09% of the flux across the rest of the body [74, 75].

In humans, this efflux of 24(S)-hydroxycholesterol corresponds to the uptake of a similar amount of 24(S)-hydroxycholesterol by the liver, which indicates the exclusive production of 24(S)-hydroxycholesterol in the brain [64]. CYP46, the cytochrome responsible for the 24S-hydroxylation of cholesterol, is localized in neurons indicating that neurons have a distinct role in the excretion of cholesterol from the brain [76] and in the retina [77]. In mice, 24S-hydroxylation of cholesterol also takes place in the liver. However, 24S-hydroxylase knockout experiments in mice revealed similar steady-state levels of cholesterol in the knockout mice and in the wild-types. In contrast to the liver, the synthesis of new cholesterol in the brain was reduced by approximately 40% in knockout animals. These data suggest that the synthesis of new cholesterol and the secretion of 24(S)-hydroxycholesterol are closely linked and that at least 40% of cholesterol turnover in the brain is dependent on the action of cholesterol 24-hydroxylase [78]. 

Liver X-receptors (LXR) LXR*α* and LXR*β* are important regulators of cholesterol homeostasis in the body. LXRs are expressed in most tissues and organs and are activated by a number of oxysterols, including 24(S)-hydroxycholesterol [79–81]. LXRs regulate their target genes including ABCA1 and ABCG1, which mediate the efflux of phospholipids and cholesterol from a number of cells including astrocytes [82], and LXR agonists have marked effects on gene expression in murine brain in a cell-specific manner. LXR agonists markedly enhance cholesterol efflux in astrocytes in culture and have only a limited effect on neuronal cultures [82]. The binding of oxysterols to LXR in the presence of large excesses of cholesterol has been questioned [83]. In the brain with a much lower ratio of oxysterols to cholesterol [67] it was postulated that a net export of cholesterol is feedback-regulated by LXR*β*, ABCA1, CYP46, and 24(S)-hydroxycholesterol [46]. LXR-dependent ABC transporters are also involved in the influx of cholesterol from (perivascular) astrocytes into the CNS [84]. Many other nuclear receptor (with ligands such as fatty acids, oxysterols, and other lipids) are also expressed in the brain. However, for most of them we still have insufficient knowledge on their role in brain development and function [85]. Also apoE, the major apo present in the CNS, is transcriptionally regulated by the ligand-activated nuclear receptors, peroxisome proliferator-activated receptor *γ*(PPAR*γ*) and LXRs [86], which form obligate heterodimers with retinoid X receptors. PPAR*γ*: retinoid X receptors and LXR: retinoid X receptors induce the expression of apoE, its lipid transporters ABCA1 and ABCG1, and the nuclear receptors itself [87]. 

## 5. Regulation of CNS Sterol Flux by the Blood-Brain Barrier

The BBB formed by tight junctions between capillary endothelial cells, separates circulating blood from the extracellular fluid in CNS. The BBB is distinct from the blood-cerebrospinal-fluid barrier, a function of the choroidal cells of the choroid plexus.

The cells of the BBB have the potential to take up low-density lipoprotein (LDL) cholesterol through luminal endothelial receptors followed by a translocation across the endothelial cell. This uptake, however, is not relevant under physiological conditions and the lipoprotein receptor-mediated uptake of cholesterol from the plasma does not regulate brain cholesterol [88]. 

This finding is supported by the normal brain phenotype of humans with LDL-receptor mutations that do not express functional LDL-receptors [89]. Most of the studies, performed on patients with pharmacological downregulation of *Hmgcr* and overexpression of LDL receptors, showed no clear effect on brain cholesterol turnover [90–92] except for one study with 18 study subjects that revealed a reduced brain cholesterol turnover [93]. 

## 6. Cholesterol and the Signaling Pathway of Hedgehog

Lipoprotein receptor (i.e., LRP2 [94]) knockout mice and holoprosencephaly caused by defects in sonic hedgehog (SHH) [95] have a very similar phenotype. Cholesterol has a pivotal role in the signaling pathway of hedgehog. SHH participates in signaling in vertebrates and avertebrates [95] and is the best studied hedgehog homologue of the 3 mammalian proteins sonic (SHH), desert (DHH), and indian (IHH). SHH consists of a ~45 kDa precursor protein and undergoes autocatalytic processing to produce an ~20 kDa N-terminal signaling domain (referred to as SHH-N) and a ~25 kDa C-terminal domain with no known signaling function. During cleavage, a cholesterol molecule is appended to the carboxyl terminus. With cholesterol attached, SHH signals in an autocrine fashion. Hedgehog signaling requires the participation of dispatched protein. When SHH has reached its target cell, it binds to the Patched (PTCH1) receptor. In the absence of a ligand, PTCH1 inhibits smoothened (SMO). SMO is regulated by a small molecule, the cellular localization of which is controlled by PTCH. An anticancer drug, vismodegib, has already been developed which interferes with SMO and has the potential to inhibit constitutively active SHH signaling pathways such as in medulloblastomas [96]. PTCH1 has homology to Niemann-Pick disease type C1 protein (NPCD1), a protein involved in the transport of lipophilic molecules across membranes. PTCH1 has a sterol sensing domain (SSD), which has been shown to be essential for the suppression of SMO activity. PTCH1 regulates SMO by removing oxysterols from SMO. For this regulation, PTCH functions like a sterol pump and removes oxysterols created by 7-dehydrocholesterol reductase. After binding to SHH protein or to the SSD of PTCH, the pump is turned off allowing the accumulation of oxysterols around SMO. The interpretation of these findings is complicated by the fact that mammals, unlike drosophila melanogaster, possess more than one hedgehog (these are SHH, DHH, and IHH) with tissue specific expression [97]. Recent data show that SHH favors the integrity during maturation of the BBB, thus providing a barrier-promoting effect and an endogenous anti-inflammatory balance to CNS-directed immune attacks [98]. 

When embryonic development is studied in mice models, the differences of the lipoprotein uptake between species has to be accounted for as well as the maturation of the BBB during embryonic development [99]. In embryonic development, cholesterol derived in the maternal organism has to cross the yolk sac endoderm to be available for the embryo. In humans, fetuses rely on maternal cholesterol supply because the endogenous synthesis is only low. Therefore, maternal cholesterol can be crucial in fetal development [100]. The contribution of maternal cholesterol to fetal cholesterol homeostasis is more prominent in rodents [101] and appears to be essential for early embryonic development in rodents, as is also evident from the lethal fetal phenotype of mice with defects in placental cholesterol transport [102]. Unlike humans, in rodents apoB and microsomal transfer protein [103] are essential for normal embryonic development. In mice, partial or total apoB deficiency limits the uptake of apoB-containing lipoproteins at the placenta and, as a consequence, these mice suffer from apparent neural tube defects [102]. Humans with apoB deficiency do not suffer from neural tube defects [8]. 

## 7. Cholesterol Synthesis Inhibitors 

Cholesterol synthesis inhibitors (CSIs) (also known as statins) are widely administered for reducing LDL cholesterol [104]. The main effect is the inhibition of *Hmgcr* but an array of pleiotropic effects of statin therapy has been observed [18, 105, 106]. Given the widespread use of statins, it is of particular interest whether CSI affect the metabolism of CNS cholesterol or not and whether an effect of (certain) CSI has any clinical impact on the CNS morphology or on neurological function [107–110]. Retrospective cohort studies have suggested that statin users have a lower prevalence of dementia. On the other hand, a randomized controlled study failed to show beneficial effects on the cognitive decline in AD [111]. 

This topic is rather complex due to several open issues. First, the CSIs used for the treatment of hypercholesterolemia (such as simvastatin, lovastatin, atorvastatin, fluvastatin, pravastatin, rosuvastatin, and pitavastatin) differ in their lipophilicity. A higher hydrophilicity/lower lipophilicity of a pharmaceutical compound and its active metabolites will, in the absence of specific transporters, lead to only very low concentrations within the CNS. Second, the very efficient mode of action of these substances, namely, the induced overexpression of LDL receptors, is not effective in the CNS since these receptors are not expressed within the CNS [89, 112]. Third, studies such as the 24S-hydroxylase knockout experiments in mice [78] indicate a very tight, unique, and independent regulation of cholesterol synthesis and efflux within and from the CNS. Finally, other effects of CSI—described as pleiotropic effects—such as effects on vascular injury, on cytokine production, and on nitric oxide production [18] might influence the integrity of the BBB [113] and any effects observed on cholesterol metabolism might be only indirect. In fact, it has been shown that simvastatin may reduce posttraumatic edema by preventing damage to tight junctions and neutrophil infiltration into the parenchyma, thus preserving BBB integrity [114].

Differences in BBB permeability coefficients might be a clue to the higher incidence of neurological adverse events of some statins. BBB permeability was studied using bovine brain microvessels or by analyzing brain extracts of statin-fed rats. Lovastatin and simvastatin had much higher BBB permeability coefficients than did fluvastatin, pravastatin, or rosuvastatin [115–117]. The results with atorvastatin and cerivastatin (the latter not available in the market anymore) are ambiguous. Some researchers report high BBB permeability coefficients for atorvastatin [118], others for cerivastatin [119]. Transfer of the lipophilic compounds lovastatin and simvastatin across the BBB occurs via passive diffusion, whereas pravastatin is taken up by an active, low affinity system [120]. The negative charge of fluvastatin affects the uptake by repulsion from the anionic microdomains in the plasma membrane of BBB endothelial cells [115]. Interesting results were obtained in the short-term treatment comparing the lipophilic simvastatin with the hydrophilic pravastatin in mice. Brain cholesterol synthesis in mice is significantly affected by simvastatin while whole-brain cholesterol turnover is not disturbed. Pravastatin can cross the BBB but does not affect intracellular cholesterol synthesis [107].

In a mechanistic approach, the effects of CSI on brain cholesterol could occur by forming a cholesterol gradient across the BBB which facilitates the efflux of cholesterol as 24(S)-hydroxycholesterol [121]. However, current knowledge is not sufficient to quantify the effect of CSI treatment on brain cholesterol. High doses (80 mg per day) of simvastatin did reduce the synthesis of CNS cholesterol *in vivo* (as analyzed by the efflux of 24(S)-hydroxycholesterol) [93] and the highly lipophilic lovastatin inhibited cholesterol synthesis and synaptogenesis *in vitro* [122]. The analysis of the ratio of plasma 24(S)-hydroxycholesterol to cholesterol after treatment of patients with high doses of CSI, however, revealed a decreased ratio and it was postulated that this was caused by a diminished substrate supply for CYP46A1 in the brain [93]. Conflicting data have been obtained from the treatment of patients with NPC disease. In NPC disease—a lysosomal lipidosis due to defective lipid transport [10]—the accumulation of tissue lipids in neuronal cells such as Purkinje cells cannot be treated by aggressive cholesterol lowering pharmaceutical approaches, neither in humans [123] nor in a mouse model [124], despite CSI therapy lowered whole body cholesterol very efficiently. When NPC disease was treated with allopregnanolone, a strong benefit was observed but it could have come solely from the vector, cyclodextrin. Cyclodextrin, a cyclical oligosaccharid with a hydrophilic exterior and a lipophilic interior is an ideal chelator for sterols and is the most effective treatment option for NPC disease in the mouse model [10]. 

Cholesterol is vital to normal brain functions including learning and memory but that involvement is as complex as cholesterol synthesis, metabolism, and excretion. Dietary cholesterol influences learning tasks in mice and rats in different experiments such as water maze and fear conditioning even though cholesterol does not cross the BBB [125, 126]. Excess cholesterol in the brain can lead to many signaling events via cholesterol metabolites, pro-inflammatory mediators, and antioxidant processes [126]. Correlations of cholesterol levels with cognitive function have been found to be positive, negative, or to have no relationship at all. An important confounder is patient age. High plasma cholesterol had detrimental effects in middle-aged persons [127–131] but had positive effects in the very elderly [132] and no effects in the young [133]. Cholesterol reduction by statin therapy improves memory in some cases but not others. Numerous reports as well as small trials have suggested that statin therapy causes cognitive impairment, although the true extent of this effect remains under study. The postmarketing adverse event reports generally described individuals over the age of 50 years who experienced notable but ill-defined memory loss or impairment that was reversible upon discontinuation of statin therapy. Time to onset of the event was highly variable, ranging from one day to years after statin exposure [105]. Muldoon et al. [134] observed minor decrements in cognitive functioning with statins. On the other end, treatment with lovastatin or pravastatin did not cause any psychological distress or substantially alter cognitive function [135, 136]. Data from the observational studies and clinical trials did not suggest that cognitive changes associated with statin use are common or that statin use leads to clinically significant cognitive decline. In any case, the FDA has recently updated the recommendation for statins in order to reduce the risk of cognitive adverse effects and to provide the public with more information about the safe and effective use of statins [137]. Although epidemiology and preclinical statin research have generally supported an adverse role of high cholesterol levels regarding Alzheimer's disease (AD), human studies of statins show highly variable outcomes, making it difficult to draw firm conclusions [105].

The difference in the lipophilicity of different statins studied was not suited to explain their fitness for use in the statin treatment of dementia or AD [138–140]. However, of particular interest are drug-specific effects obtained in gene expression studies when comparing different CSI [141]. Interesting results were also observed when studying the effects of statins in the presence and absence of mevalonate. Despite cholesterol synthesis was blocked by the inhibition of *Hmgcr*, as expected, the isoprenylation continued unimpeded [142]. From the current knowledge, the differing effects of different statins on brain functions might be modulated by their effects on metabolic pathways other than the cholesterol pathway.

Hypercholesteremia can induce *τ*-hyperphosphorylation and A*β* production in rat brain. Atorvastatin inhibited *τ*-hyperphosphorylation and decreased A*β* generation, thus playing a protective role in the pathogenesis of hypercholesteremia-induced neurodegeneration in the brain [143]. In mice, early treatment with both atorvastatin and pitavastatin prevented subsequent worsening of cognitive function and the amyloidogenic process, probably due to pleiotropic effects, suggesting a therapeutic potential for AD patients [144]. In AD patients, simvastatin treatment caused a modest but significant inhibition of brain cholesterol biosynthesis, as measured by a decrease of cerebrospinal fluid lathosterol and plasma 24(S)-hydroxycholesterol. Despite these effects, there were no changes in AD biomarkers [145]. In another study, atorvastatin was not associated with significant clinical benefit [146]. One possible interpretation might be that statins prevent or delay the onset of AD, but cannot slow the cognitive decline once the disease process has started.

Higher levels of isoprenoids favor APP-processing by *α*-secretase and less amyloid *β* (A*β*) is being secreted. Low concentrations of isoprenoids inhibit the metabolism of APP through the secretory pathway and lead to the intracellular accumulation of A*β*. Low intracellular cholesterol levels inhibit receptor-mediated endocytosis of APP. In mice, the inhibition of protein isoprenylation by fluvastatin, at a clinically relevant dose, reduced brain A*β* levels by increasing the trafficking of APP carboxyl terminal fragments and by enhancing A*β* clearance mediated by upregulation of low-density lipoprotein receptor-related protein 1 (LRP-1) expression [147].

Other protective effects of statins besides their plasma cholesterol lowering without alteration of the isoprenoid levels [142] were obtained in studies with stroke patients and in prospective studies. In observational studies, patients under CSI treatment had lower likelihoods of mortality as well as of poor functional outcome [148] and the discontinuation of CSI after ischemic stroke has been associated with worse outcomes [149]. Studies with very high doses (up to 8 mg/kg body weight) of lovastatin are under way and have indicated an acceptable safety profile [150]. The use of CSI, however, has been shown to increase the risk of bleeding after ischemic stroke, irrespective of plasma cholesterol concentration. Studies show no clear positive effects for CSI on cerebral hemorrhage, although atorvastatin has been shown to significantly relieve brain edema, to decrease the brain injury caused by matrix metalloproteinase-9, and to protect neurons in rats with intracerebral hemorrhage [151]. 

Other important confounding factors of the human epidemiological studies performed so far are the stage of Alzheimer's disease in the patients studied, the drug dose, the duration of therapy and the adherence to the prescribed drug, and the overlap of Alzheimer's disease with other forms of dementia and vascular disease [105]. While *in vitro* effects are fairly well understood, the results from *in vivo* studies in humans are conflicting and additional research work is urgently needed with highly standardized study designs to address the impact of CSI on brain cholesterol metabolism and Alzheimer's disease. Currently, routine use of CSI in CNS disease cannot be recommended except for lowering LDL cholesterol in cerebrovascular disease [106].

## 8. Cerebrospinal Fluid Lipoproteins

Essentially all cholesterol within the CNS is associated with cell membranes and only tiny amounts are located within the intercellular space and in the CSF under physiological conditions. Cholesterol within the intercellular space and in the CSF is associated with apos, in particular with apoE. A very high expression of apoE was observed during regeneration of peripheral nerves after damage [152] and apoE—associated with lipids—was the most abundant protein in the intracellular fluid. ApoE is also expressed within the CNS and astrocytes secrete lipoprotein particles composed of apoE, apoAI, and lipids (Figure 1). ApoE in the CNS has a higher apparent molecular weight due to a higher sialylation and is exclusively lipid-bound [14, 153]. The role of apoE-containing lipoproteins is postulated to redistribute lipids and to regulate cholesterol homeostasis within the brain.

The concentration of lipoproteins in the CSF is low. Compared to plasma, in CSF the concentration of apoE is about 5%, of cholesterol about 1%, and of phospholipids about 2% only. Triglycerides are present in trace amounts and apoB is absent. The concentration of lipids in the CSF does not correlate with the plasma lipid concentration [154]. CSF lipoproteins are bigger in size than plasma HDL and smaller than LDL, their density is between LDL and HDL [14]. Their composition with esterified cholesterol as core lipid, their size, and density is very similar to lipoproteins secreted from macrophages [155] or from transfected neuro2a cells [156]. However, nascent apoE/lipid particles secreted by cultured astrocytes are primarily discoid and contain only small amounts of lipids such as cholesterol and phospholipids [157]. Besides small amounts of apoA-I, CSF lipoproteins contain trace amounts of apoA-II, apoC-I, and apoC-III. These small, lipophilic apos are derived from the plasma and have leaked through the blood brain barrier. ApoE and apoB cannot cross the BBB [158], however, in case of a BBB breakdown, plasma components can spill into the CSF and apoB can be detected in the CSF.

## 9. LDL Receptor Family in the CNS

Numerous lipoprotein receptors of the LDL receptor family have been detected in the CNS by biochemical and molecular methods (LDL receptor, VLDL-receptor, apoER2/LRP8, LRP4, LRP, LRP2 (formerly known as gp330 or megalin), LRP1B, LRP5/LRP6, and LRP11/SORL1) [13, 159]. Ligands for these receptors are apoE-containing lipoproteins, lipids and other macromolecules [14, 153, 160]. However, the spatial expression in different cells such as neurons, astrocytes and microglia—even different for cell lines within different regions of the brain—and the different temporal expression with a particular high expression postnatally suggest important functions during embryogenesis and for the functioning of the CNS. In fact, these multifunctional and evolutionary ancient receptors have been shown to be intercellular signal transducers and signal modulators [13].

The differential roles of these receptors in the CNS are impeded by their redundant functions, similar or even identical specificity for certain ligands, and the coexpression in certain organs or even in one cell. Transgenic animal models are of very limited use because of relevant interspecies differences in particular in regard of structural differences of the placenta and the necessity of certain lipoproteins and lipoprotein receptors for the lipid transfer from the maternal organism during embryogenesis. The requirement for the same receptor at two stages of lipid transport into the embryo makes it difficult to unequivocally determine the participation of these molecules at a certain step, that is, on the yolk sac surface or at the neuroepithelium [37]. 

The study of the evolutionary highly conserved signaling pathways by mouse genetics revealed critical functions for the receptors of the apoE-receptor family. Both knockout mice lacking LRP2 [161] and double knockout mice lacking both apoER2/LRP8 and the VLDL-receptor [162] show severe defects in brain development. The latter are phenotypically indistinguishable from mice lacking the signaling protein Reelin and from mice with mutations in the cytoplasmic adapter protein Disabled-1 (*Dab1*) [163]. This phenotype suggested that apoER2/LRP8, as well as the VLDL-receptor, functions in a linear signaling pathway that is dependent on the extracellular ligand Reelin and the intracellular adaptor Dab1 for initiating a signaling cascade that regulates the migration and positioning of neurons during development [163]. Further studies revealed a signaling through the Src family tyrosine kinase through proximity triggered phosphorylation [164]. Other signaling pathways are also active in microglia and neurons as indicated by the interaction of the ATP-binding cassette transporter ABCA1 (the member 1 of human transporter subfamily ABCA), also known as cholesterol efflux regulatory protein (CERP), with the MAP-kinase-system [165].

The novel downstream interactions are partly dependent upon unique interaction motifs within the cytoplasmic domain of the receptors which helps to explain the specific and nonredundant roles of ER2/LRP8 and VLDL-receptor in shaping brain regions in response to binding of the same ligand. For instance, the specific topography of the Purkinje cell layer is regulated by the ER2/LRP8 and the VLDL-receptor and in the neocortex [166], the VLDL-receptor provides a stop signal for migrating neurons while apoER2/LRP8 regulates the migration of later-borne neurons essential for the proper lamination of neocortex and hippocampus [162]. In the peripheral neurons, LRP4 is essential for the function of the neuromuscular junction. LRP4-deficient mice are stillborn. LRP4 is an obligate coreceptor for the muscle specific tyrosine kinase MUSK and the neuronally produced ligand agrin. Only in the presence of LRP4, agrin leads to clustering and transphosphorylation of MUSK. In the absence of LRP4, MUSK transphosphorylation cannot occur and acetylcholine receptors fail to form postsynaptic clusters [167].

Despite all genes of the LDL receptor family have been disrupted in the mouse, human mutations besides the LDL receptor have been identified so far only in LRP2 and in the VLDL receptor. Defects in LRP2 cause Donnai-Barrrow syndrome, congenital agenesis of the corpus callosum, diaphragmatic hernia, facial dysmorphology, ocular anomalies, sensorineural hearing loss, and development delay [168]. The defects in patients are similar but not identical to those seen in LRP2 knockout C57bl/6X129 hybrid mice [168].This indicates genetic modifiers among species and even within different mice strains. VLDL-receptor deficiency, a fully autosomal recessive trait, leads to a similar but more severe phenotype in humans compared to mice [169]. In humans, distinct mutations have been observed in the Canadian Hutterite population [170] and in Middle Eastern populations [171]. Neuroanatomically, these mutations are indistinguishable but considerable variations exist in regard of motor development. Several, but not all, affected individuals are quadrupedal and it has been suggested that the VLDL receptor is the critical gene for the evolution of the bipedal gait [171]. Other human autosomal recessive diseases with virtually identical phenotypes to VLDL receptor deficiency are caused by mutations of chromosome 17 [172]. Crk, a downstream component of the Reelin signaling pathway, is located in this chromosome region [169].

## 10. Apolipoproteins (apoE and apoJ) within the CNS

The pivotal role of apoE for cholesterol recycling in the regenerating peripheral nerve has been long recognized [173]. ApoE, a 39 kD protein, is the major apo in the CNS. Three major alleles of *Apoe* (*ε*2, *ε*3, *ε*4) are expressed codominantly and lead to common phenotypes E22, E23, E24, E33, E34, and E44. *ε*3 is the most common allele (about 77%) and *ε*2 is the least common (about 8%) of these major alleles [174]. ApoE2 has a defective binding to the LDL-receptor and is responsible for type III hyperlipidemia. ApoE3 as well as apoE4 are high affinity ligands for the LDL-receptor [175]. ApoE consists of two domains (the N- and C-terminal domains) separated by a protease-sensitive hinge region. The differences between apoE isoforms are arginine-to-cysteine changes in the N-terminal domain. ApoE4 contains arginines at positions 112 and 158, whereas apoE3 has a Cys112 [175]. 

The functions of apoE in the CNS are heterogeneous and range from participation in cholesterol homeostasis and in nonlipid activities such as protein chaperoning and signal transduction [176]. ApoE is expressed in the brain in high concentrations, such that the brain is the organ with the second highest apoE expression after the liver [177]. Astrocytes are the major source of apoE followed by oligodendrocytes, microglia, and ependymal layer cells [178]. The significance of apoE expression in neurons has not been resolved yet. Some CNS neurons may express apoE under certain condition such as after excitotoxic injury [179] and apoE expression was observed in primary cultures of human hippocampal neurons and in mice expressing human apoE under the control of the human apoE promoter [176]. The stability of apoE in the brain requires the association with lipids. In knockout mice for Abca1—a gene necessary for the lipidation of apoE—the amount of apoE in the brain is reduced [180, 181]. The LDL-receptor and LRP1 are the main receptors for the uptake of apoE containing lipoprotein particles in the brain. LDL-receptor knockout mice have increased levels of apoE in brain parenchyma and in CSF [182] which suggests impaired metabolism of apoE. A conditional deletion of *Lrp1 *gene in mouse brain decreases brain parenchyma apoE [182]. ApoE is the major apo of CSF lipoproteins [14, 157, 158]. Similar to lipoprotein cholesterol, apoE does not cross the BBB. This was demonstrated by the analysis of apoE phenotypes after liver transplantation when no donor apoE can be found in the CSF of the recipient [177]. Whether apoE has a major role in supporting synaptogenesis and maintenance of synaptic connections *in vivo* in the uninjured brain is unclear. In apoE knockout mice, brain morphology and behavior are not altered in the absence of injury [183, 184] and no overt cognitive defects have been reported in humans with genetic apoE deficiency [185]. It has been suggested that there is a dynamic exchange of apoE among brain cells, that apoE is the major transport protein for extracellular cholesterol and other lipids, and that apoE-mediated cholesterol exchange occurs between neuronal and nonneuronal cells of the CNS [176].

Several lines of evidence link apoE with late-onset Alzheimer's disease, the most common cause of dementia in the elderly. Epidemiological studies highlighted the *ε*4 allele of *Apoe* as the most common risk factor for late-onset Alzheimer's disease [186], and *in vitro* studies showed a colocalization of apoE with amyloid plaques [187] and a positive correlation between plaque density and number of *ε*4 alleles in Alzheimer' patients at autopsy [188].

ApoE is a risk factor with relatively low penetrance but with high prevalence [189]. Individuals with one *ε*4 allele are 3 to 4 times more likely than those without *ε*4 alleles to develop late-onset Alzheimer's disease [189] and suffer from Alzheimer's disease at a younger age [190]. Individuals with the *ε*2 allele have a lower risk of late-onset Alzheimer's disease compared to the *ε*3 allele ([186], for results of meta-analyses see http://www.alzgene.org/). However, most homozygotes for *ε*4 will not suffer from Alzheimer's disease and most patients with Alzheimer's disease are not homozygous for *ε*4 [189]. 

The search for the molecular mechanisms of apoE4 promoting Alzheimer's disease is still ongoing despite 20 years of intense research. These molecular mechanisms would be attractive pharmaceutical targets for the prevention and treatment of Alzheimer's disease [176]. One hypothesis proposes a “toxic” mechanism of apoE4, the other hypothesis is a failure of apoE4 to slow down the disease process as effectively as apoE3 and apoE2 do. These hypotheses have direct implications in devising therapeutic strategies. In the first hypothesis, the treatment would have to block apoE4, in the second hypothesis the treatment has to potentiate or to mimic apoE3 [191]. 

Biochemical studies elucidated isoform-specific differences between different apoE isoforms, in particular between apoE3 and apoE4. The design of these studies is hindered by the lack of apoE isoforms in animals. Different apoE isoforms are only present in humans; all animal apoEs studied so far contain Thr at the equivalence to the position Arg61 in human apoE4. Therefore, irrespective of the amino acid at position 112, animal apoE behaves like human apoE3 [192]. 

ApoE4 binds to large lipoprotein particles, attributable to the presence of Arg112, which affects the conformation of the side chain of Arg61 in the N-terminal domain resulting in domain interaction between Arg61 and Glu255 in the C-terminal domain [193]. Unlike apoE3, apoE4 is assumed to produce an unstable “molten globule” state that is responsible for the pathogenic role in Alzheimer's disease [194]. Molten globule domains interact with different target molecules, typically with high-affinity and low specificity [195]. Since the isoform-specific differences between apoE3 and apoE4 are outside the receptor-binding region, studies have evaluated the molecular interaction of apoE with proteins present in the neuritic plaque. Lipid-bound (i.e., physiologic) as well as artificially delipidated apoE forms complexes with A*β*. However, the order of stability of the complexes dependent on apoE isoform was not uniform [196, 197]. Data from *in vitro *studies indicate that apoE enhances cellular A*β* uptake and degradation [196, 198, 199] but *in vivo* studies revealed that apoE retards A*β* clearance [200, 201], possibly via an effect at the BBB [202]. Until now, the whole wild type apoE could not be crystallized and all crystallographic studies could only be performed on fragments of the protein or after site directed mutagenesis [203]. Another challenge results from self-aggregation of the protein and from the formation of oligomers. When lipids are added to the delipidated protein, the protein undergoes large structural changes [204], and it is not clear whether differences observed with the delipidated protein are retained after lipidation [203]. The structure of the N-terminal domain (i.e., the receptor binding domain) of apoE3 and apoE4 has been determined using both X-ray and NMR methods [205, 206]. The isolated C-terminal domain—responsible for lipid binding, for oligomerization and probably for A*β* binding—is known to aggregate which prevents structure determination by standard methods [192]. Studies performed with transgenic animals only expressing apoE 3 or apoE4 either in neurons or in astrocytes showed a detrimental effect of apoE4 on all subunits of mitochondrial respiratory complexes assessed and treatment of these cells with a compound disrupting apoE4 domain interaction restored mitochondrial respiratory complex IV levels. Mutant apoE4 (apoE4-Thr-61) lacking domain interaction did not induce mitochondrial dysfunction [207]. 

Recently, results of the first pharmacological interventions with apoE as target performed in mice have been reported. One study has targeted at the apoE isoform-specific effects on the BBB. In the absence of murine apoE, human apoE4 but not apoE3 or apoE2 leads to BBB breakdown by activating the proinflammatory cyclophilin A in pericytes [208]. Another study overexpressed (murine) apoE in the brain by the oral application of the FDA-approved RXR agonist bexarotene. This study demonstrated a beneficial effect of CNS apoE overexpression [209], and it was concluded that apoE overexpression might be able to decrease the progression of A*β* deposition. The proposed mechanism consists in the absorption of A*β* by the CNS-derived lipoproteins followed by the clearance of these particles from the brain. However, only murine apoE was tested and no apoE isoform-specific effects have been reported so far [191]. Another study employed an apoE-mimetic peptide called ApoE-(133-149) which was administered intraperitoneally, has optimized BBB penetration, and showed neuroprotection [210]. Previous studies with this peptide in mice with targeted replacement of apoE indicate human apoE isoform-specific effects on immune reactions with an increase in proinflammatory cytokines in apoE4-expressing mice [211]. Unlike native apoE, ApoE-(133-149) lacks the lipid binding region and will not be exclusively lipid-bound. Besides its immunomodulatory effects on microglia in the CNS and macrophages outside the CNS, the peptide may affect cholesterol metabolism as well as LRP expression and LRP receptor binding [211]. 

Apolipoprotein J (also known as clusterin) is also present in lipoprotein particles and regulates cholesterol and brain lipid metabolism [212]. Similar to apoE, the expression of clusterin in the brain changes significantly during development [213] and during different kinds of neuronal injuries [214] and increases in Alzheimer's disease [212]. Clusterin is a versatile chaperone molecule which contains several amphipathic and coiled-coil alpha-helices, typical characteristics of small heat shock proteins. Clusterin was originally identified in Sertoli cells but very early, the high expression of clusterin in the brain [215] and its role in cholesterol trafficking has been established [212]. The secondary structure of clusterin contains molten globule domains [216]. Of particular interest is the role of clusterin in the clearance of A*β* peptides. Secreted A*β* peptides bind avidly to apoE and to clusterin/apoJ, which prevents the oligomerization of these A*β* peptides. A minor part of this complex is endocytosed by microglia and astrocytes and degraded by insulin degrading enzyme and neprilysin but the major part is endocytosed without degradation. The major receptors for the uptake at the blood-brain barrier are LRP and LRP2 [94, 217]. The third clearance pathway, besides the proteolytic degradation and the endocytic uptake by microglia and astrocytes, is the efflux of A*β* peptides through the blood-brain barrier. This efflux is mediated by LRP1 [218] and by LRP2 [219]. A downregulation of LRP1 is correlated with regional A*β* accumulation in Alzheimer's disease [218]. For the clearance of A*β* in the brain, apoE and clusterin/apoJ have a comparable and interchangeable function. 

In summary, the function of the apoE receptors in neurons is distinct but with similar molecular mechanisms, in particular in intra- and intercellular signaling. These receptors are critical for the formation and the function of central and of peripheral synapses. Cholesterol and apoE play a particular role as constituents of the ligands to these receptors.

## 11. Amyloid Precursor Protein and Cholesterol

The accumulation, oligomerization, and deposition of A*β* (a cleavage product of amyloid precursor protein APP) are hallmark of Alzheimer's pathogenesis. A*β* is a intrinsically disordered protein, which can self-aggregate and form an array of supramolecular assemblies with different morphology including oligomers, amorphous aggregates, and amyloid like fibrils. Cholesterol is involved in several steps of A*β* processing. The elimination products, the oxysterols such as the brain-specific 24(S)-hydroxycholesterol, as well as 27-hydroxycholesterol increase sAPP production and contribute to amyloidogenesis [220] with 24(S)-hydroxycholesterol having more pronounced effects than 27-hydroxycholesterol [81]. Plasma 27-hydroxycholesterol concentration was associated with CSF sAPP levels [221]. 

The selective expression of CYP46A1 around neuritic plaques and the potent inhibition of APP processing in neurons by 24(S)-hydroxycholesterol suggest that CYP46A1 affects the pathophysiology of Alzheimer's disease [81]. ApoE and its receptors are closely involved in these processes. ApoE as well as LRP1 are present in amyloid plaques [187] and apoE regulates isoform-specifically APP processing to A*β* by mechanisms dependent on LRP1 function. ApoE4 increases A*β* production in neuronal cells overexpressing APP to a greater extent than apoE3. After incubation with the LRP1 receptor antagonist RAP or by RNA interference, this isoform-specific effect was blunted [222]. Three other apoE receptors (LRP1B, APOER2, LRP11/SORL1) also interact with APP and regulate its trafficking and processing to A*β* [223]. All these receptors reduce A*β* processing due to their slow rate of endocytosis. In the absence of common ligands, however, APOER2 increases the distribution of APP into special cholesterol-rich membrane subdomains (also known as detergent-resistant membrane fraction (DRM) or lipid rafts) and the processing of APP to A*β* [224]. DRMs are abundant on the cell surface and are enriched in cholesterol and sphingolipids and a variety of proteins. Sorting signals that target proteins to DRMs include binding to cholesterol, sphingolipids and GPI-anchors, and other lipid modifications such as palmitoylation and myristoylation [3]. Lipid-lipid interactions are one of the major mechanisms to form DRMs. DRMs are thought to represent liquid-ordered (L_O_) domains, which coexist in the same membrane together with liquid-disordered (L_d_) domains [3]. L_O_ domains are formed due to the preferential interaction of sphingolipids and/or phospholipids containing saturated acyl chains with cholesterol [225]. Cholesterol has been shown to be a critical regulator of DRM formation and the local regulation of cholesterol levels by cholesterol biosynthesis, compartmentalization and secretion of cholesterol is a powerful mechanism to control DRM-dependent events. 

A*β* is cleared from the brain by two pathways. First, through receptor-mediated endocytosis by cells in brain parenchyma and along the interstitial fluid or through the BBB and second, through endopeptidase-mediated proteolytic degradation. The receptor-mediated endocytosis by members of the LDL-receptor family on astrocytes [226] and microglia [227] can be an efficient way to reduce brain A*β*. Most A*β* internalized by apoE receptors is degraded in lysosomes or is transcytosed into the plasma. However, A*β* can escape from degradation or transcytosis, accumulates in neurons, and exerts its toxicity on the neuronal function [228]. Opposite to murine studies in which apoE overexpression decreased A*β* deposition [208–210], in mice overexpressing A*β* under control of the PDGF promoter, the lack of apoE markedly diminished A*β* deposition [229].

A*β* generation is localized in DRMs or rafts. The enzyme responsible for the enzymatic cleavage of APP called *β*-secretase, or *β*-site APP cleaving enzyme (BACE), is found in DRMs in primary hippocampal neurons [230]. The other proteins necessary for the processing of APP have been located in the DRMs of the late Golgi and endosomes, the organelles where BACE is thought to be biologically activated. The complete A*β* generating proteolytic machinery colocalizes within DRMs of the same vesicle [231]. A clue to the complex APP processing comes from studying the effects of different cholesterol concentrations [230]. In cells being cholesterol-depleted and overexpressing APP, further lowering of cholesterol inhibits amyloidogenesis [91, 232, 233]. In neurons with normal cholesterol content, a moderate reduction of cholesterol led to increased A*β* levels whereas a strong cholesterol reduction resulted in a significant drop in A*β* generation. These cholesterol dose-dependent effects can be explained by two independent cellular mechanisms [231]. First, a moderate reduction of cholesterol causes a disorganization of DRMs allowing more BACE to interact with APP and to generate A*β*. A strong reduction in cholesterol inhibits BACE/*γ*-secretase activities. Despite a direct contact of BACE/*γ*-secretase with APP, A*β* generation is blunted. In cells overexpressing APP, APP can be mislocalized in DRMs and then cleaved by BACE [230, 233]. 

## 12. Conclusion

The pivotal effects of brain cholesterol on different cellular processes in the CNS have become apparent in the last decades. Most of these studies were performed in patients with inherited defects of cholesterol biosynthesis and in transgenic animals with defined mutations. The intriguing data on the substantial role of cholesterol in the physiology and pathophysiology of DRMs has been predominantly obtained in cell culture studies. 

While many studies have focused on the cholesterol metabolism of the whole CNS, late work focused on cell-specific and even microdomain-specific effects of cholesterol. Human and animal studies are complicated by different expression of genes involved during development as well as by cell specific differences. Another challenge is the role of the BBB, which also changes during development and can be regulated under certain pathological conditions. The sterol flux between CNS cells (namely neurons and oligodendrocytes) is only poorly understood. However, whole CNS cholesterol production can be very elegantly studied by analyzing the concentrations of 24(S)-hydroxycholesterol, the exclusive metabolite of CNS cholesterol. Studies of the hedgehog signaling cascade in humans were driven by the SLO syndrome and this disease could be clarified by studying the genes involved in human patients as well as in transgenic mice models. Still under discussion are the options of treatment of CNS diseases by targeting CNS cholesterol metabolism. Differences in pharmacology, as well in the selection of patients, obscure potential beneficial or detrimental effects and it is currently not recommended to treat patients with CSI for these indications outside from clinical trials. 

The analysis of CSF lipoproteins revealed some data on brain cholesterol metabolism. However, no clinical applicable tests have been developed so far which employ CSF lipoproteins or apos for the clinical diagnosis or for monitoring the effects of certain therapies. Studies of the LDL receptor family in the CNS showed a very interesting redundancy as well as interaction of these lipoprotein receptors with a wide array of important signal transduction pathways. While these receptors are capable of taking up lipoproteins, this function is only one of many others involved in cell differentiation, growth, and function. Isoform-specific effects of apoE have been the bridge to the processing of APP. A great number of studies have addressed the rather complicated molecular processing and the effects of cholesterol (both in the whole organism as well as in single cells) on this processing. From current understanding, CNS cholesterol is an auspicious target for preventing or even treating Alzheimer's disease. The full understanding of these process remains a huge challenge given the increasing number of older patients, the very complex pharmacokinetics of drugs targeting the CNS, the long time course of presymptomatic disease, and many confounding factors identified so far.

## Abbreviations

A*β*: Amyloid *β*
apo: Apolipoprotein
APP: Amyloid precursor protein
BACE: *β*-site APP cleaving enzyme
BBB: Blood brain barrier
CNS: Central nervous system
CSF: Cerebrospinal fluid
CSI: Cholesterol synthesis inhibitors
*Dhcr*7: 7-dehydrocholesterol reductase
DRM: Detergent-resistant membrane fraction
Hmgcr: 3-hydroxy-3-methylglutaryl-coenzyme A reductase
L_d_: Liquid-disordered domain
LDL: Low-density lipoprotein
L_o_: Liquid-ordered domain
LXR: Liver X-receptors
NPC: Niemann-Pick type C
NPC1: Niemann-Pick disease type C1 protein
PPAR*γ*: Peroxisome proliferator-activated receptor *γ*
PTCH1: Patched
RAGE: Receptor for advanced glycation end products
SHH: Sonic hedgehog
SMO: Smoothened.
